# The quantitative impact of prostate-specific membrane antigen (PSMA) PET/CT staging in newly diagnosed metastatic prostate cancer and treatment-decision implications

**DOI:** 10.1093/bjro/tzae040

**Published:** 2024-11-08

**Authors:** Hoda Abdel-Aty, Nabil Hujairi, Iain Murray, Yathushan Yogeswaran, Nicholas van As, Nicholas James

**Affiliations:** Division of Radiotherapy and Imaging, Institute of Cancer Research, London, SW3 6JB, United Kingdom; Department of Radiotherapy, Royal Marsden NHS Foundation Trust, London, SW3 6JJ, United Kingdom; Division of Radiotherapy and Imaging, Institute of Cancer Research, London, SW3 6JB, United Kingdom; Department of Radiology and Nuclear Medicine, Royal Marsden NHS Foundation Trust, London, SM2 5PT, United Kingdom; Division of Radiotherapy and Imaging, Institute of Cancer Research, London, SW3 6JB, United Kingdom; Department of Radiology and Nuclear Medicine, Royal Marsden NHS Foundation Trust, London, SM2 5PT, United Kingdom; MRC Clinical Trials Unit, Institute of Clinical Trials & Methodology, University College London, London, WC1V 6LJ, United Kingdom; Division of Radiotherapy and Imaging, Institute of Cancer Research, London, SW3 6JB, United Kingdom; Department of Radiotherapy, Royal Marsden NHS Foundation Trust, London, SW3 6JJ, United Kingdom; Division of Radiotherapy and Imaging, Institute of Cancer Research, London, SW3 6JB, United Kingdom; Department of Radiotherapy, Royal Marsden NHS Foundation Trust, London, SW3 6JJ, United Kingdom

**Keywords:** oligometastatic disease, polymetastatic disease, PET/CT, prostate-specific membrane antigen, stereotactic ablative body radiotherapy

## Abstract

**Objectives:**

To quantify the stage-shift with prostate-specific membrane antigen (PSMA) PET/CT imaging in metastatic prostate cancer and explore treatment implications.

**Methods:**

Single-centre, retrospective analysis of patients with newly diagnosed [^18^F]PSMA-1007 or [^68^Ga]Ga-PSMA-11 PET/CT-detected metastatic prostate cancer who had baseline bone scintigraphy between January 2015 and May 2021. Patients were subclassified into oligometastatic and polymetastatic disease utilizing the STAMPEDE2 trial (ISRCTN66357938/NCT06320067) definition. Patient, tumour, and treatment characteristics were collected. PSMA PET/CT concordance with conventional imaging (bone scintigraphy and low-dose CT of PET) was identified by number and site of metastases, and subgroup assigned. Spearman’s rank correlation and linear regression modelling determined the association between the imaging modalities.

**Results:**

We analysed 62 patients with a median age was 72 years (range 48-86). On PSMA PET/CT, 31/62 (50%) patients had oligometastatic disease, and 31/62 (50%) had polymetastatic disease. Prostate radiotherapy was delivered in 20/31 (65%) patients with oligometastatic disease and 17/31 (55%) with polymetastatic disease. 23/62 (37%) patients were reclassified as M0 on conventional imaging. PSMA PET/CT had a 2.9-fold increase in detecting bone metastases. Bone metastases concordance was found in 10/50 (20%) by number and 30/33 (91%) by site. PSMA PET/CT had a 2.2-fold increase in detecting nodal metastases. Nodal metastases concordance was found in 5/46 (11%) by number and 25/26 (96%) by site. There was significant positive correlation between PSMA PET/CT and conventional imaging for detecting bone [*R*^2^ = 0.25 (*P *<* *0.001)] and nodal metastases [*R*^2^ = 0.19 (*P *<* *0.001)]. 16/31 (52%) had oligometastatic disease concordance.

**Conclusion:**

The magnitude of PSMA PET/CT-driven stage-shift is highly variable and unpredictable with implications on treatment decisions, future trial design, and potentially clinical outcomes.

**Advances in knowledge:**

The magnitude of “frame-shift” with PSMA PET/CT imaging is highly variable and unpredictable which may unreliably change treatment decisions dependent on image-defined disease extent. Prospective randomized trials are required to determine the relationship between PSMA PET/CT-guided treatment choices and outcomes.

## Introduction

Traditionally, conventional imaging with CT and bone scintigraphy is standard of care for prostate cancer staging. However, conventional imaging is limited by suboptimal detection of loco-regional and metastatic disease.^1–3^ The preferential overexpression of prostate-specific membrane antigen (PSMA) protein has become the fore of interest for its use as a target for imaging with radio-labelled molecules collectively referred to as PSMA PET/CT.^4^ The proPSMA study was the largest, prospective randomized trial to report on the superior accuracy of PSMA PET/CT compared to conventional imaging for the initial staging of prostate cancer.^5^ Moreover, the role of PSMA PET/CT in biochemically recurrent disease has been established^6–8^ with subsequent integration of PSMA PET/CT imaging in international guidelines.^9^^,^^10^

PSMA PET/CT therefore offers a more sensitive means of detecting low volume metastatic disease. This is pertinent to treatment approaches relying on defining disease extent. In synchronous oligometastatic disease, prostate radiotherapy has shown overall survival benefit.^11^^,^^12^ Additionally, promising results for metastasis directed therapy with stereotactic ablative body radiotherapy (SABR) have been demonstrated in metachronous oligometastatic disease.^13–17^ PSMA PET/CT in these settings may potentially influence clinical decision-making, though the impact on overall outcomes is unknown. Most studies have focussed on the detection of occult metastatic disease in intermediate- and high-risk loco-regional prostate cancer.

In this study, we quantitatively analyse the stage-shift with PSMA PET/CT compared to conventional imaging staging in newly diagnosed PSMA PET/CT-detected metastatic prostate cancer and review the potential impact of this disease stage shift informing treatment decisions.

## Materials and methods

### Study population

We reviewed the first PSMA PET/CT imaging performed for all patients with any prostate cancer disease stage at our institution. Men with newly diagnosed PSMA PET/CT detected metastatic prostate cancer who had a baseline bone scintigraphy within 182 days (6 months) of the PSMA PET/CT were analysed. The baseline PSMA PET/CT had to be carried out within 182 days (6 months) of commencing androgen deprivation therapy (ADT).

Data on patient and tumour characteristics at time of diagnosis were collected and included: patient’s age, presenting prostate-specific antigen (PSA), the International Society of Urological Pathology (ISUP) grade group, and tumour staging defined using the TNM 8th edition staging system.

Data were collected on treatment given included use of ADT, additional systemic therapy with docetaxel or androgen receptor pathway inhibitor (ARPI) (or both), and external beam radiotherapy.

### Subgroup classification

Patients were classified into oligometastatic and polymetastatic disease subgroups on PSMA PET/CT and conventional imaging. Oligometastatic disease was defined as 5 or fewer bone and/or extra-pelvic (non-regional) lymph node metastases. Polymetastatic disease was defined as more than 5 bone and/or extra-pelvic lymph node metastases, or the presence of visceral metastases. The subgroup definition was based on the STAMPEDE2 trial (ISRCTN66357938/NCT06320067) definition.

### Imaging acquisition

#### PSMA PET/CT imaging

PSMA PET/CT images were acquired on a PET/CT scanner (Gemini TF; Philips or Biograph mCT; Siemens) 60 min after the injection of a 150 MBq [^68^Ga]Ga-PSMA-11, or 250 MBq [^18^F]PSMA-1007 respectively. Data were acquired either for 2 min ([^18^F]PSMA-1007) or 3 min ([^68^Ga]Ga-PSMA-11) per bed position. Both Biograph mCT and Gemini TF PSMA PET data were reconstructed using 3-dimensional ordered subsets expectation maximization algorithm incorporating time-of-flight.^18^

#### Conventional imaging

Conventional imaging was denoted by the cross-sectional imaging from the low-dose CT component of the PSMA PET/CT and bone scintigraphy. For both PSMA PET/CT scanners, low-dose CT imaging was performed for localization and attenuation correction. Parameters were 120 kVp, reference mAs of 50 mAs, 3 mm slice width and separation. For bone scintigraphy, whole-body dual headed planar gamma camera imaging was performed approximately 3 h after administration of ^99m^Tc labelled phosphonate using an Intveo (Siemens Healthineers) system with the following acquisition parameters: 140 ± 7.5% keV photopeak, 256 × 1024 matrix, 20 cm per minute speed, low energy high-resolution collimators.

#### Multiparametric MRI prostate and pelvis

Multiparametric MRI (mpMRI) was carried out at 3 T or 1.5 T (Magnetom Skyra or Sola systems, Siemens Healthineers Erlangen). Standard protocols include axial T1-weighted images of the pelvis along with axial and coronal T2-weighted small field of view images of the prostate, axial diffusion weighted imaging (b50, b600m b1050, and calculated b1400) and corresponding apparent diffusion coefficient map. Dynamic contrast enhanced imaging was with a Dixon gradient echo sequence following the injection of 0.2 mL/kg gadolinium at 3 mL/s and temporal resolution of <15 s.

### Image interpretation

Interpretation of the baseline PSMA PET/CT, bone scintigraphy and mpMRI images was performed through retrospective review of the clinical imaging reports and/or documented review at centralized multi-disciplinary team meetings. The low-dose CT images were independently reviewed by a dual trained radiologist and nuclear medicine physician, blinded to the PSMA PET/CT imaging report.

### Imaging concordance

Concordance of PSMA PET/CT with mpMRI was determined for T (tumour) and N (nodal) disease stage. Concordance of PSMA PET/CT with conventional imaging was determined for M (metastases) disease stage for bone, nodal, visceral metastases, and subgroup assigned. Per-patient concordance on both modalities was defined by:

- The same number of metastases detected.
- The same site of metastases detected.
- The same subgroup assigned.

The total count of metastases was determined for each of bone and nodal metastases on PSMA PET/CT and conventional imaging. Patients with 10 or more metastases were placed in a single grouping. The detection ratio for bone metastases between both modalities was determined accounting for metastases considered equivocal on conventional imaging as negative or positive.

### Statistical analysis

Concordance was reported using descriptive analysis. The per-patient correlation between PSMA PET/CT and conventional imaging for nodal and bone metastases was examined utilizing the Spearman’s rank correlation coefficient and linear regression modelling to determine the relationship between the number of metastases on each imaging modality. For the correlation analysis, equivocal bone metastases on conventional imaging were considered negative. Statistical analysis was performed using Stata statistical software version 17.0 (StataCorp, College Station, TX, USA).

The study was carried out as a retrospective audit. Access to collected data was approved by the Committee for Clinical Research and Development at our institution (Reference SE1111).

## Results

### Study population

Between January 2015 and May 2021, 2023 patients had PSMA PET/CT imaging at any stage of their prostate cancer diagnosis. We excluded 1782 patients with PSMA PET/CT confirmed intermediate-risk localized disease (*n* = 74), high-risk localized disease (*n* = 641), castrate-resistant disease (*n* = 144), recurrent disease (*n* = 919), and subsequent non-prostate cancer diagnosis (*n* = 4). Of the remaining 241 patients, we excluded 179 with newly diagnosed prostate cancer who had baseline PSMA PET/CT only. Our analysis consisted of 62 patients.

**Figure 1.**
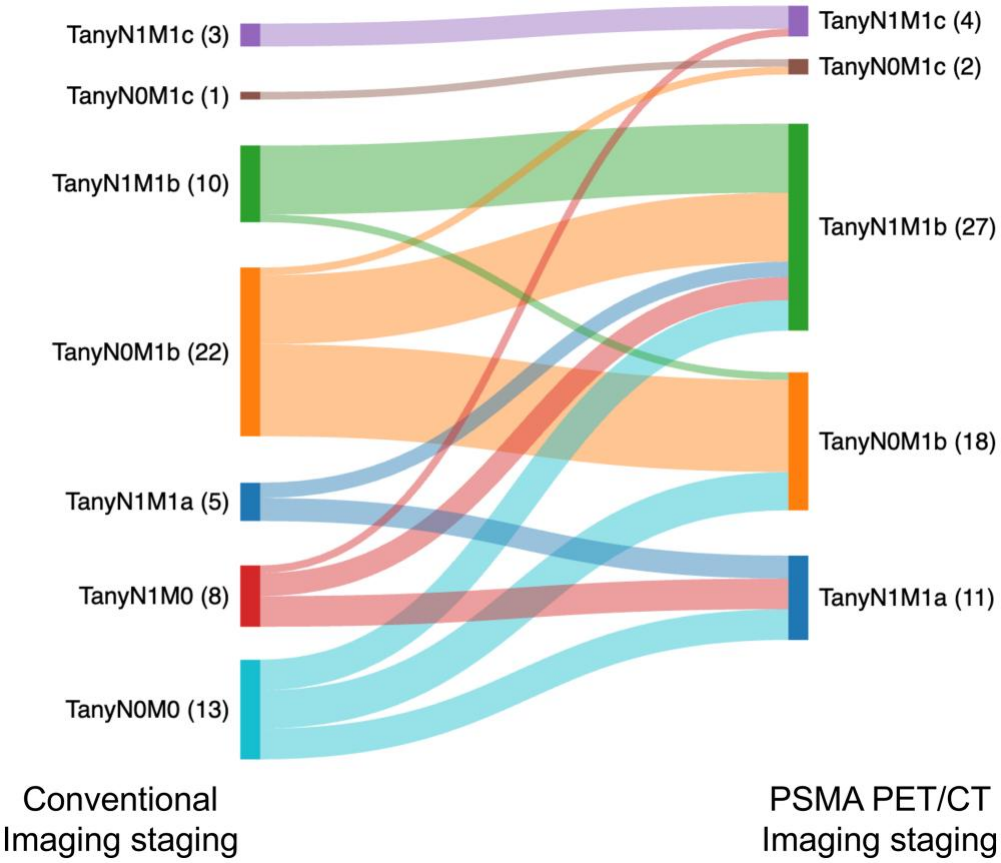
Sankey plot demonstrating the TNM stage shift between conventional imaging and PSMA PET/CT staging and the potential impact on treatment decision change. Standard of care at the time of diagnosis for this cohort for stage T any N any M0 was radical treatment with up to 3 years of androgen deprivation therapy (ADT) and prostate ± loco-regional radiotherapy. For stage T any N any M1a was long-term ADT, additional systemic therapy with docetaxel or androgen receptor pathway inhibitors (ARPIs) and prostate only radiotherapy. For stage T any N any M1b was long-term ADT, additional systemic therapy and prostate only radiotherapy if 3 or fewer bone metastases were confirmed on conventional imaging. For stage T any N any M1c standard of care treatment was long-term ADT and additional systemic therapy.

The median age at presentation was 72 years [range 48-86, interquartile range (IQR) 66-76], and the median PSA was 42 ng/mL (range 4.8-5400, IQR 17-100). The most common ISUP Grade group was group 5 in 44/62 (71%) patients. T3a or higher disease was identified in 58/62 (94%) patients on mpMRI prostate and pelvis, and 54/62 (87%) on PSMA PET/CT. T stage concordance between mpMRI prostate and pelvis and PSMA PET/CT was observed in 42/62 (68%) patients. Pelvic nodal disease was detected in 34/62 (55%) on mpMRI prostate and pelvis, and in 42/62 (68%) patients on PSMA PET/CT. N stage concordance between mpMRI prostate and pelvis and PSMA PET/CT was observed in 51/62 (82%) patients. Table 1 summarizes tumour characteristics and TNM staging on mpMRI, PSMA PET/CT, and bone scintigraphy imaging modalities. The significant TNM staging shift between conventional imaging and PSMA PET/CT imaging is demonstrated in Figure 1.

**Table 1.** Patient and tumour characteristics at baseline.

Tumour characteristics		*N* (%)
ISUP Grade Group	Non-histological diagnosis	4 (6)
	Group 2	4 (6)
	Group 3	8 (13)
	Group 4	2 (3)
	Group 5	44 (71)
**mpMRI TNM stage**		
T stage	T1a-T2c	4 (6)
	T3a	13 (21)
	T3b	30 (48)
	T4	15 (24)
N stage	Nx	1 (2)
	N0	27 (43)
	N1	34 (55)
M stage	Mx	36 (58)
	Equivocal M1b	10 (16)
	M1a	1 (2)
	M1b	15 (24)
**PSMA PET/CT TNM stage**		
T stage	Tx	2 (3)
	T1a-T2c	6 (10)
	T3a	17 (27)
	T3b	30 (48)
	T4	7 (11)
N stage	N0	20 (32)
	N1	42 (68)
M stage	M1a	11 (18)
	M1b^a^	45 (72)
	M1c^b^	6 (10)
**Bone scintigraphy**		
M stage	M0	29 (47)
	M1b	33 (53)

a Includes 18/45 (40%) patients with M1a disease (extra-pelvic nodal metastases) in addition to M1b (bone metastases) simultaneously.

b Includes 3/6 (50%) patients with M1a disease (extra-pelvic nodal metastases), and 5/6 (83%) patients with M1b disease (bone metastases) in addition to M1c disease (visceral metastases) simultaneously.

The type of PSMA PET/CT imaging performed were [^68^Ga]Ga-PSMA-11 in 44/62 (71%) and [^18^F]PSMA-1007 in 7/62 (11%).

The median interval time between the baseline bone scintigraphy and PSMA PET/CT was 30 days (IQR 18-45). The median interval time between commencing ADT and the baseline PSMA PET/CT scan was 18 days (IQR 8-39). A total of 46/62 (74%) patients had commenced ADT prior to PSMA PET/CT scan with a median time of 20 days (IQR 14-41).

### Bone metastases

**Figure 2.**
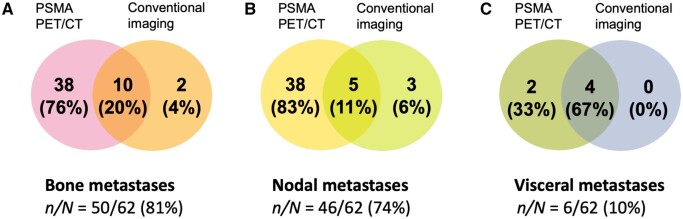
Venn diagrams showing the per-patient concordance of PSMA PET/CT with conventional imaging based on the detected number of (A) bone, (B) nodal (pelvic and extra-pelvic), and (C) visceral metastases. 10/50 (20%) patients had the same number of bone metastases, 5/46 (11%) had the same number of nodal metastases, and 4/6 (67%) had the same number of visceral metastases detected on both imaging modalities.

Bone metastases were detected in 50/62 (81%) patients on PSMA PET/CT, and in 33/62 (53%) on conventional imaging. Concordance by the number of bone metastases detected for each imaging modality is shown in Figure 2A.

**Figure 3.**
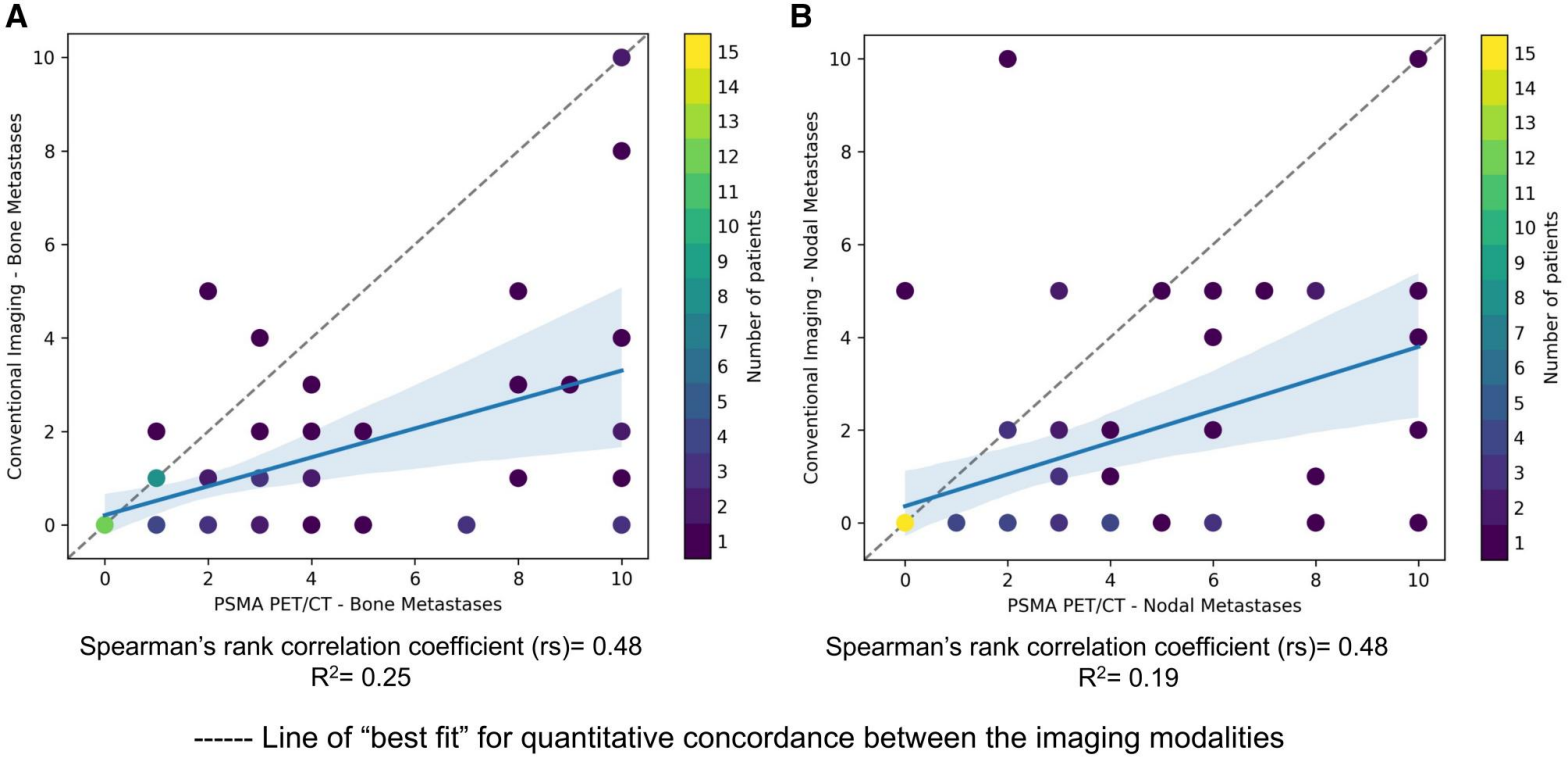
Scatterplot demonstrating the correlation between PSMA PET/CT and conventional imaging for detecting (A) bone metastases and (B) nodal metastases with positive correlation between the imaging modalities.

Across all patients, 230 bone metastases were detected on PSMA PET/CT, and 79 bone metastases were detected on conventional imaging. When equivocal bone metastases were considered negative, the detection ratio was 2.9:1 in favour of PSMA PET/CT. When equivocal bone metastases were considered positive, 84 bone metastases were detected on conventional imaging, resulting in a detection ratio of 2.7:1 in favour of PSMA PET/CT. Concordance by the site of bone metastases for each imaging modality was present in 30/33 (91%) patients. Results of the Spearman’s rank correlation suggested evidence of a significantly positive correlation between PSMA PET/CT and conventional imaging for detecting bone metastases [rs = 0.48 (95% CI 0.27-0.65, *P *<* *0.001), *R*^2^= 0.25 (95% CI 0.004-0.47, *P *<* *0.001)] (Figure 3A).

### Nodal metastases

Pelvic and/or extra-pelvic nodal metastases were present in 46/62 (74%) patients on PSMA PET/CT, and in 26/62 (42%) patients on conventional imaging. Concordance in the number of nodal metastases identified by each imaging modality is shown in Figure 2B.

Across all patients, PSMA PET/CT detected 209 nodal metastases and conventional imaging detected 94 nodal metastases, resulting in a detection ratio of 2.2:1 for nodal metastases in favour of PSMA PET/CT.

Concordance by the site of nodal metastases for each imaging modality was present in 25/26 (96%) patients. Results of the Spearman’s rank correlation suggested evidence of a significantly positive correlation between PSMA PET/CT and conventional imaging for detecting nodal metastases [rs = 0.48 (95% CI 0.26-0.65, *P *<* *0.001), *R*^2^ = 0.19 (95% CI 0.05-0.42, *P *<* *0.001)] (Figure 3B).

### Visceral metastases

Visceral metastases were present in 6/62 (10%) patients on PSMA PET/CT, and 4/62 (67%) on conventional imaging (Figure 2C). 2/6 (33%) patients with visceral metastases detected on PSMA PET/CT only had penile shaft metastases.

### Subgroup classification

**Figure 4.**
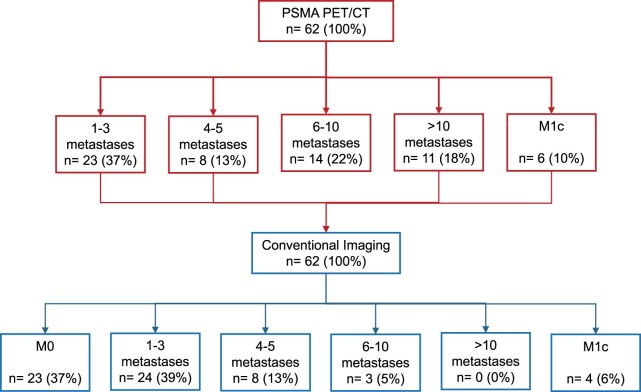
CONSORT diagram. Subgroup classification on PSMA PET/CT and reclassification on conventional imaging.

On PSMA PET/CT imaging, 31/62 (50%) patients had oligometastatic disease, 31/62 (50%) had polymetastatic disease. On conventional imaging, 23/62 (37%) patients had M0 disease, 32/62 (52%) had oligometastatic disease, and 7/62 (11%) had polymetastatic disease. Figure 4 shows the subgroup classification for each imaging modality.

Table 2 summarizes the treatments given for the entire cohort and for each subgroup based on PSMA PET/CT staging.

**Table 2.** Treatment characteristics guided by PSMA PET/CT staging.

Treatment characteristics	Oligometastatic disease	Polymetastatic disease	All cohort
***n*/*N* (%)**	**31/62 (50)**	**31/62 (50)**	**62 (100)**
ADT	31 (100)	31 (100)	62 (100)
Additional systemic treatment^a^			
Docetaxel	11 (36)	10 (32)	21 (34)
Abiraterone	7 (23)	6 (19)	13 (21)
Enzalutamide	11 (36)	9 (29)	20 (32)
Apalutamide	0 (0)	1 (3)	1 (2)
None	5 (16)	6 (19)	11 (18)
External beam radiotherapy			
Prostate only	14 (45)	14 (45)	28 (45)
Prostate and pelvic LN	3 (10)	1 (3)	4 (6)
Prostate, pelvic LN and BM	3 (10)	1 (3)	4 (6)
Prostate and BM	0 (0)	1 (3)	1 (2)
SABR to BM^b^	1 (3)	0 (0)	1 (2)
Palliative radiotherapy	1 (3)	3 (10)	4 (6)
None	9 (29)	11 (36)	20 (32)

a 4/62 (6%) patients switched between additional systemic therapies due to treatment-related toxicity.

b This one patient received SABR to metastases in addition to prostate and pelvic LN radiotherapy.

Abbreviations: ADT = androgen deprivation therapy; BM = bone metastases; LN = lymph nodes.

**Figure 5.**
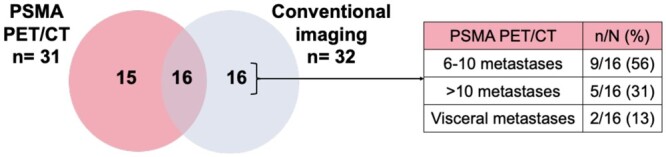
Concordance of PSMA PET/CT with conventional imaging in oligometastatic disease. PSMA PET/CT confirmed oligometastatic disease in 16/32 (50%) patients. Of the remaining, 16/32 (50%) with oligometastatic disease on conventional imaging, PSMA PET/CT confirmed polymetastatic disease with up to 10 metastases in 9/16 (56%) patients.

Oligometastatic disease concordance of PSMA PET/CT with conventional imaging is shown in Figure 5.

Among the 31/62 (50%) patients with polymetastatic disease on PSMA PET/CT, 14/31 (45%) patients had 6 to 10 metastases. Of those, 9/14 (64%) had oligometastatic disease, and 4/14 (29%) had M0 disease on conventional imaging.

**Figure 6.**
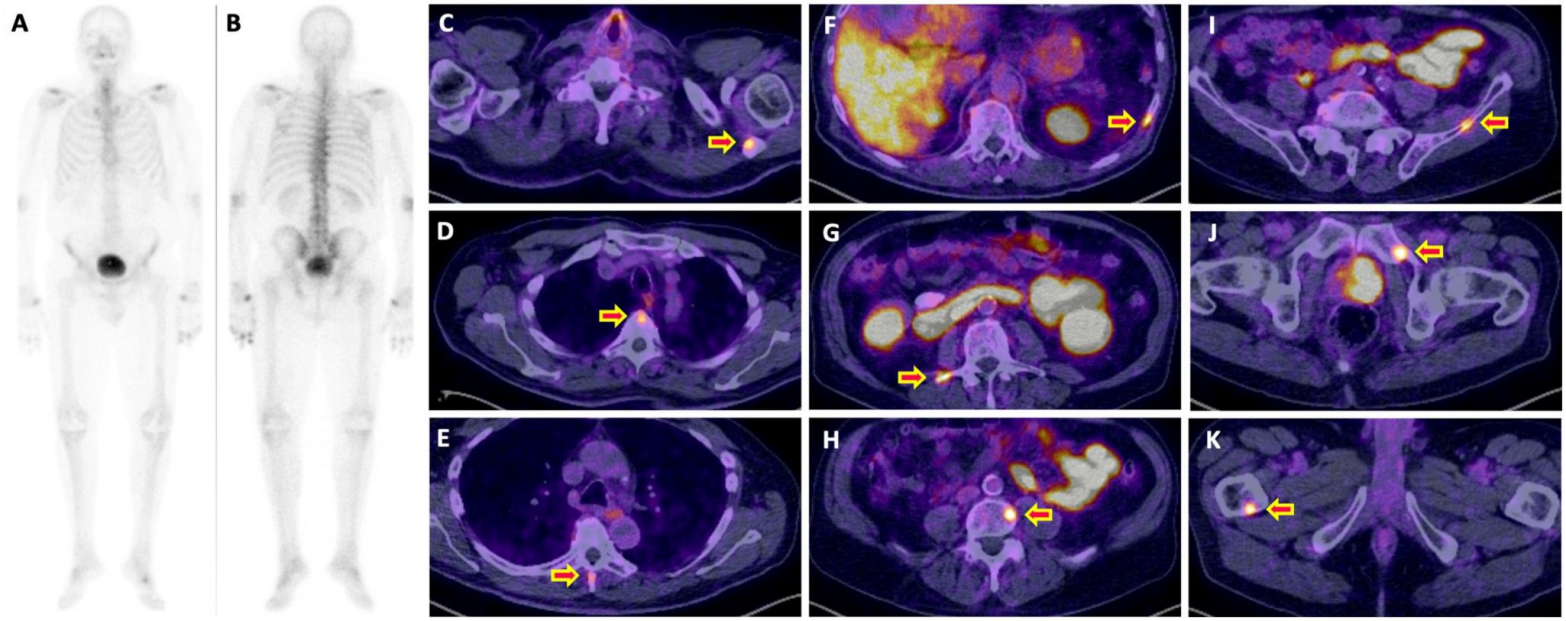
Example patient with “right-stage” shift with PSMA PET/CT staging. Seventy-seven-year-old patient, Gleason score 4 + 5 = 9 (ISUP grade group 5), presenting PSA 15 ng/mL, and T3b disease with equivocal left superior pubic ramus lesion on MRI (not shown in figure). (A and B) Baseline bone scintigraphy with SPECT/CT (not shown in figure) reported non-specific degenerative changes. (C-K) [^68^Ga]Ga-PSMA-11 PET/CT performed 30 days from bone scintigraphy showed the primary tumour and multiple bone metastases in (C) left scapula, (D, E, G, H) thoraco-lumbar spine (F) ribs, (I-J) pelvic bones, (K) right femur. Abbreviations: ISUP = International Society of Urological Pathology; PSA = prostate-specific antigen; PSMA = prostate-specific membrane antigen; SPECT/CT = single-photon emission computed tomography/CT.

11/31 (36%) had polymetastatic disease on PSMA PET/CT, with more than 10 metastases. Of those, 2/11 (18%) patients had polymetastatic disease, 5/11 (45%) patients had oligometastatic disease, and 4/11 (36%) patients had M0 disease on conventional imaging. Figure 6 demonstrates an example patient with M0 disease on conventional imaging reclassified with polymetastatic disease on PSMA PET/CT imaging.

## Discussion

PSMA PET/CT imaging in prostate cancer allows potential treatment modification. This study assessed the impact of PSMA PET/CT on treatment decisions in the metastatic setting, focussed around the M0/M1 boundary on conventional imaging where the potential for management change is greatest. Our results demonstrated a significant PSMA PET/CT-driven “right-shift” in disease stage with 37% of patients reclassified as M0 on conventional imaging. The study confirmed superior detection rates of PSMA PET/CT for bone and nodal metastases, corroborating existing literature.^5–8^ The stage reclassification in our study with PSMA PET/CT imaging has significantly influenced clinical decisions. We observed variability in systemic therapy choices and delivery of external beam radiotherapy between subgroups. This highlights the challenges associated with the uncertainty when applying current evidence-based treatment to PSMA PET/CT staged disease and how it should be modified, as it undoubtably is.

[^68^Ga]Ga-PSMA-11 and [^18^F]PSMA-1007 PET/CT imaging were both included in our analysis given their comparable clinical impact^19^ and interchangeable recommendations in international clinical guidelines,^9^^,^^10^ despite reported differences in their production, biodistribution, spatial resolution, clinical accessibility,^20^ and higher rates of bone false positivity with [^18^F]PSMA-1007 which could be circumvented by experienced reporting clinicians.^21^

Most therapeutic trials have traditionally relied on conventional imaging for treatment evidence.^22^ Current international guidelines recommend prostate radiotherapy in men with low burden metastatic disease on conventional imaging^9^^,^^10^ defined by the CHAARTED criteria.^23^ This is based on results from the STAMPEDE M1RT comparison which showed significant survival gains.^11^^,^^12^ Secondary analyses indicate the effectiveness of prostate radiotherapy beyond 3 bone metastases,^24^ with quantitative bone metastatic burden predictive of response.^25^

PSMA PET/CT staging may reclassify patients with low-burden metastatic disease as having widespread metastases, potentially leading to the omission of prostate radiotherapy. In M0 disease, where the survival benefit from prostate radiotherapy is known,^26^^,^^27^ the role of PSMA PET/CT remains under investigation. Current evidence, derived from non-randomized studies has shown that PSMA PET/CT detects distant metastases in 6%-9% of patients, with 13%-20% requiring treatment modification, such as radiotherapy dose escalation or pelvic lymph node irradiation.^28–32^ However, PSMA PET/CT could also risk under-dosing or omission of radiotherapy. The optimal treatment approach for these groups, whether based on conventional or PSMA PET/CT staging, remains unclear.

First described in 1995,^33^ oligometastatic disease definitions varied across trials.^11^^,^^23^^,^^34^ We defined oligometastatic disease utilizing the STAMPEDE2 trial (ISRCTN66357938/NCT06320067), derived from secondary analyses of the STAMPEDE M1RT comparison,^24^ to align with published consensus classifications.^35^^,^^36^ Studies have demonstrated the role of SABR in metachronous oligometastatic prostate cancer, identified through various imaging modalities.^13–17^ In the ORIOLE trial, SABR showed the greatest benefit when delivered to all PSMA PET/CT detected metastases.^14^ Conversely, the higher sensitivity of PSMA PET/CT in detecting early metastases may result in “lead-time bias,” persuading clinicians to potentially overtreat patients who may otherwise have indolent disease with excellent outcomes.^37^ Additionally, the emerging evidence of “triplet therapy” with docetaxel in addition to ARPI also means that volume of metastatic disease is a potential driver for treatment intensification in some patients, exposing some patients to avoidable long-term side effects.^38^^,^^39^

**Figure 7.**
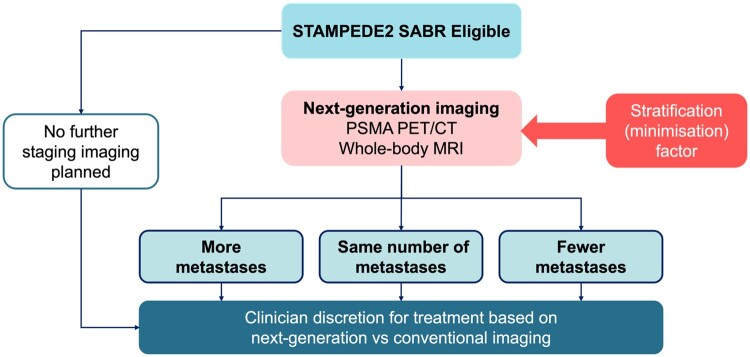
STAMPEDE2 SABR trial (Comparison S) next generation imaging sub-study. Abbreviations: PSMA PET/CT = prostate-specific membrane antigen PET/CT; SABR = stereotactic ablative body radiotherapy.

The utility of PSMA PET/CT imaging poses significant implications on future trial recruitment as extrapolation of current evidence to the PSMA PET/CT era remains unclear. Prospective clinical trials should consider the implementation of standardized reporting frameworks to objectively interpret PSMA PET/CT imaging.^40^ A recent UK-based survey indicated that many sites do not use PSMA PET/CT for initial staging, suggesting heterogenous practice is likely to remain for the medium-term pending construction of new imaging capacity.^41^ Similar variations exist internationally.^42^ The STAMPEDE2 SABR trial (ISRCTN66357938/NCT06320067) will test SABR in synchronous oligometastatic disease defined by conventional imaging. An embedded next generation imaging sub-study will aim to bridge the evidence gap between the imaging modalities as shown in Figure 7.

In our study, 50% of patients with oligometastatic disease on conventional imaging were reclassified as polymetastatic disease on PSMA PET/CT. This observed shift confirms how treatment decisions are critically dependent on imaging and the concept of oligometastatic disease is elastic, heavily reliant on imaging, significantly impacting what is otherwise a simple trial question.

Our results showed bone metastases detection was 2.9 times higher with PSMA PET/CT than conventional imaging. The high concordance at the lesion level implies that PSMA PET/CT scans are detecting the same metastatic sites and additional ones. However, and very importantly, while the overall ratio of PSMA PET/CT to conventional metastasis detection was 2-3, the range of ratios was broad and inconsistent, suggesting that a simple “frame-shift” model is not feasible, thus the relationship of conventional imaging to PSMA PET/CT stage is unpredictable.

The dichotomous association between androgen signalling and PSMA expression suggests potential up- or down-regulation of PSMA expression within a few days of commencing ADT and ARPI.^43–45^ Our findings may indicate upregulation of PSMA, explained by the increased number of metastases detected, given most of our patients (74%) had commenced ADT at the time of PSMA PET/CT scan. Further studies are needed to evaluate the complexity of this association to accurately assess the most appropriate time for PSMA PET/CT imaging and understand the implications of different treatments on PSMA expression.

The limitations of the study include its retrospective design, dependence on imaging reports and reviews from multi-disciplinary team meetings for baseline PSMA PET/CT and bone scintigraphy, and the absence of histopathological confirmation of metastases. We analysed the non-contrast low-dose CT due to the unavailability of contrast-enhanced staging CT. The low-dose CT was utilized to assess for nodal metastases which might be sufficient as suggested by Marchetti et al.^46^ However, we acknowledge this is not the ideal scenario and may not be fully informative. To minimize this limitation, the low-dose CT was independently reviewed by an experienced radiologist. Finally, the inclusion of patients with metastatic disease on conventional imaging with negative PSMA PET/CT scans was beyond the scope of the study.

Our study has documented the magnitude and inconsistency of PSMA PET/CT-driven stage shift. PSMA PET/CT imaging will inevitably present treatment decision challenges for clinicians and patients as it is yet to be determined whether the application of known conventional imaging-based treatments will be validated when applied to potentially different disease stages.^47^ Additionally, the reclassification of patients and interpretation of oncological outcomes can result in apparent outcome improvements that are artefactual—the Will Rogers phenomenon.^48^

## Conclusion

PSMA PET/CT imaging has higher detection rate for metastases over conventional imaging in metastatic prostate cancer. The substantial, but variable, PSMA PET/CT-driven magnitude of “right stage” shift observed had a significant impact on treatment decision-making with unpredictable consequences. Future work within prospective randomized clinical trials should integrate cross comparison of the imaging modalities with standardized PSMA PET/CT reporting frameworks to ascertain the true impact of PSMA PET/CT imaging on clinical outcomes.

## References

[1] Hövels AM , Heesakkers RAM, Adang EM, et al The diagnostic accuracy of CT and MRI in the staging of pelvic lymph nodes in patients with prostate cancer: a meta-analysis. Clin Radiol. 2008;63(4):387-395. 10.1016/J.CRAD.2007.05.022 18325358

[2] Rowe SP , Macura KJ, Ciarallo A, et al Comparison of prostate-specific membrane antigen-based 18F-DCFBC PET/CT to conventional imaging modalities for detection of hormone-naive and castration-resistant metastatic prostate cancer. J Nucl Med. 2016;57(1):46-53. 10.2967/jnumed.115.163782 26493203 PMC4730886

[3] Hu X , Cao Y, Ji B, Zhao M, Wen Q, Chen B. Comparative study of 18F-DCFPyL PET/CT and 99mTc-MDP SPECT/CT bone imaging for the detection of bone metastases in prostate cancer. Front Med (Lausanne). 2023;10:1201977. 10.3389/fmed.2023.1201977 37588003 PMC10425766

[4] Kiess AP , Banerjee SR, Mease RC, et al Prostate-specific membrane antigen as a target for cancer imaging and therapy. Q J Nucl Med Mol Imaging. 2015;59(3):241-268. 26213140 PMC4859214

[5] Hofman MS , Lawrentschuk N, Francis RJ, et al; proPSMA Study Group Collaborators. Prostate-specific membrane antigen PET-CT in patients with high-risk prostate cancer before curative-intent surgery or radiotherapy (proPSMA): a prospective, randomised, multicentre study. Lancet. 2020;395(10231):1208-1216. 10.1016/S0140-6736(20)30314-7 32209449

[6] Fendler WP , Calais J, Eiber M, et al Assessment of 68Ga-PSMA-11 PET accuracy in localizing recurrent prostate cancer: a prospective single-arm clinical trial. JAMA Oncol. 2019;5(6):856-863. 10.1001/JAMAONCOL.2019.0096 30920593 PMC6567829

[7] Pienta KJ , Gorin MA, Rowe SP, et al A phase 2/3 prospective multicenter study of the diagnostic accuracy of prostate specific membrane antigen PET/CT with 18F-DCFPyL in prostate cancer patients (OSPREY). J Urol. 2021;206(1):52-61. 10.1097/JU.0000000000001698 33634707 PMC8556578

[8] Morris MJ , Rowe SP, Gorin MA, et al; CONDOR Study Group. Diagnostic performance of 18 F-DCFPyL-PET/CT in men with biochemically recurrent prostate cancer: results from the CONDOR Phase III, Multicenter Study. Clin Cancer Res. 2021;27(13):3674-3682. 10.1158/1078-0432.CCR-20-4573 33622706 PMC8382991

[9] Schaeffer EM , Srinivas S, Adra N, et al NCCN guidelines^®^ insights: prostate cancer, version 3.2024: featured updates to the NCCN guidelines. J Natl Compr Canc Netw. 2024;22(3):140-150. 10.6004/jnccn.2024.0019 38626801

[10] EAU Guidelines on Prostate Cancer—Uroweb. Uroweb—European Association of Urology n.d. 2024. Accessed July 20, 2024. https://uroweb.org/guidelines/prostate-cancer

[11] Parker CC , James ND, Brawley CD, et al The Radiotherapy to the primary tumour for newly diagnosed, metastatic prostate cancer (STAMPEDE): a randomised controlled phase 3 trial. Lancet. 2018;392(10162):2353-2366. 10.1016/S0140-6736(18)32486-3 30355464 PMC6269599

[12] Parker CC , James ND, Brawley CD, et al STAMPEDE Trial Collaborative Group. Radiotherapy to the prostate for men with metastatic prostate cancer in the UK and Switzerland: Long-term results from the STAMPEDE randomised controlled trial. PLoS Med. 2022;19(6):e1003998. 10.1371/JOURNAL.PMED.1003998 35671327 PMC9173627

[13] Ost P , Reynders D, Decaestecker K, et al Surveillance or metastasis-directed therapy for oligometastatic prostate cancer recurrence: a prospective, randomized, multicenter phase II trial. J Clin Oncol. 2018;36(5):446-453. 10.1200/JCO.2017.75.4853 29240541

[14] Phillips R , Shi WY, Deek M, et al Outcomes of observation vs stereotactic ablative radiation for oligometastatic prostate cancer: the ORIOLE Phase 2 Randomized Clinical Trial. JAMA Oncol. 2020;6(5):650-659. 10.1001/jamaoncol.2020.0147 32215577 PMC7225913

[15] Chalkidou A , Macmillan T, Grzeda MT, et al Stereotactic ablative body radiotherapy in patients with oligometastatic cancers: a prospective, registry-based, single-arm, observational, evaluation study. Lancet Oncol. 2021;22(1):98-106. 10.1016/S1470-2045(20)30537-4 33387498

[16] Palma DA , Olson R, Harrow S, et al Stereotactic ablative radiotherapy for the comprehensive treatment of oligometastatic cancers: long-term results of the SABR-COMET Phase II randomized trial. J Clin Oncol. 2020;38(25):2830-2838. 10.1200/JCO.20.00818 32484754 PMC7460150

[17] Harrow S , Palma DA, Olson R, et al Stereotactic radiation for the comprehensive treatment of oligometastases (SABR-COMET): extended long-term outcomes. Int J Radiat Oncol Biol Phys. 2022;114(4):611-616. 10.1016/J.IJROBP.2022.05.004 35643253

[18] Popescu LM , Matej S, Lewitt RM. Iterative image reconstruction using geometrically ordered subsets with list-mode data. In: *IEEE Symposium Conference Record Nuclear Science*, Rome, Italy. Vol. 6. 2004:3536-3540. 10.1109/NSSMIC.2004.1466649

[19] Huang S , Ong S, McKenzie D, et al Comparison of 18F-based PSMA radiotracers with [68Ga]Ga-PSMA-11 in PET/CT imaging of prostate cancer—a systematic review and meta-analysis. Prostate Cancer Prostatic Dis. 2024;654-664. 10.1038/s41391-023-00755-2 38017295 PMC11543591

[20] Maisto C , Aurilio M, Morisco A, et al Analysis of pros and cons in using [68Ga]Ga-PSMA-11 and [18F]PSMA-1007: production, costs, and PET/CT applications in patients with prostate cancer. Molecules (Basel, Switzerland). 2022;27(12):38-62. 10.3390/MOLECULES27123862 PMC9227284 35744985

[21] Seifert R , Telli T, Opitz M, et al Unspecific 18F-PSMA-1007 bone uptake evaluated through PSMA-11 PET, bone scanning, and MRI triple validation in patients with biochemical recurrence of prostate cancer. J Nucl Med. 2023;64(5):738-743. 10.2967/jnumed.118.215434 36460340

[22] Hussain M , Carducci MA, Clarke N, et al Evolving role of prostate-specific membrane antigen-positron emission tomography in metastatic hormone-sensitive prostate cancer: more questions than answers? J Clin Oncol. 2022;40(26):3011-3014. 10.1200/JCO.22.00208 35439030

[23] Sweeney CJ , Chen Y-H, Carducci M, et al Chemohormonal therapy in metastatic hormone-sensitive prostate cancer. N Engl J Med. 2015;373(8):737-746. 10.1056/NEJMoa1503747 26244877 PMC4562797

[24] Ali A , Hoyle A, Haran ÁM, et al Association of bone metastatic burden with survival benefit from prostate radiotherapy in patients with newly diagnosed metastatic prostate cancer: a secondary analysis of a randomized clinical trial. JAMA Oncol. 2021;7(4):555-563. 10.1001/jamaoncol.2020.7857 33599706 PMC7893550

[25] Ali A , Hoyle AP, Parker CC, et al; STAMPEDE Investigators. The automated bone scan index as a predictor of response to prostate radiotherapy in men with newly diagnosed metastatic prostate cancer: an exploratory analysis of STAMPEDE’s “M1\RT comparison”. Eur Urol Oncol. 2020;3(4):412-419. 10.1016/j.euo.2020.05.003 32591246 PMC7443695

[26] Widmark A , Klepp O, Solberg A, et al; Swedish Association for Urological Oncology 3. Endocrine treatment, with or without radiotherapy, in locally advanced prostate cancer (SPCG-7/SFUO-3): an open randomised phase III trial. Lancet. 2009;373(9660):301-308. 10.1016/S0140-6736(08)61815-2 19091394

[27] Warde P , Mason M, Ding K, et al; NCIC CTG PR.3/MRC UK PR07 Investigators. Combined androgen deprivation therapy and radiation therapy for locally advanced prostate cancer: a randomised, phase 3 trial. Lancet. 2011;378(9809):2104-2111. 10.1016/S0140-6736(11)61095-7 22056152 PMC3243932

[28] Nikitas J , Lam E, Booker KA, et al Randomized trial of prostate-specific membrane antigen PET/CT before definitive radiotherapy for unfavorable intermediate- and high-risk prostate cancer (PSMA-dRT trial). J Nucl Med. 2024;65(7):1076-1079. 10.2967/jnumed.123.267004 38664019 PMC11218723

[29] Frenzel T , Tienken M, Abel M, et al The impact of [68Ga]PSMA I&T PET/CT on radiotherapy planning in patients with prostate cancer. Strahlenther Onkol. 2018;194(7):646-654. 10.1007/s00066-018-1291-5 29572670

[30] Roach PJ , Francis R, Emmett L, et al The impact of 68 Ga-PSMA PET/CT on management intent in prostate cancer: results of an Australian Prospective Multicenter Study. J Nucl Med. 2018;59(1):82-88. 10.2967/JNUMED.117.197160 28646014

[31] Calais J , Kishan AU, Cao M, et al Potential impact of 68Ga-PSMA-11 PET/CT on the planning of definitive radiation therapy for prostate cancer. J Nucl Med. 2018;59(11):1714-1721. 10.2967/jnumed.118.209387 29653978 PMC6225538

[32] Hruby G , Eade T, Emmett L, et al 68 Ga-PSMA-PET/CT staging prior to definitive radiation treatment for prostate cancer. Asia Pac J Clin Oncol. 2018;14(4):343-346. 10.1111/ajco.12872 29663686

[33] Hellman S , Weichselbaum RR. Oligometastases. J Clin Oncol. 1995;13(1):8-10. 10.1200/JCO.1995.13.1.8 7799047

[34] Baciarello G , Özgüroğlu M, Mundle S, et al Impact of abiraterone acetate plus prednisone in patients with castration-sensitive prostate cancer and visceral metastases over four years of follow-up: a post-hoc exploratory analysis of the LATITUDE study. Eur J Cancer. 2022;162:56-64. 10.1016/J.EJCA.2021.11.026 34953443

[35] Guckenberger M , Lievens Y, Bouma AB, et al Characterisation and classification of oligometastatic disease: a European Society for Radiotherapy and Oncology and European Organisation for Research and Treatment of Cancer consensus recommendation. Lancet Oncol. 2020;21(1):e18-e28. 10.1016/S1470-2045(19)30718-1 31908301

[36] Lievens Y , Guckenberger M, Gomez D, et al Defining oligometastatic disease from a radiation oncology perspective: an ESTRO-ASTRO consensus document. Radiother Oncol. 2020;148:157-166. 10.1016/J.RADONC.2020.04.003 32388150

[37] Sutera P , Song Y, Van der Eecken K, et al Clinical and genomic differences between advanced molecular imaging-detected and conventional imaging-detected metachronous oligometastatic castration-sensitive prostate cancer. Eur Urol. 2023;84(6):531-535. 10.1016/j.eururo.2023.04.025 37173210 PMC10636237

[38] Fizazi K , Foulon S, Carles J, et al; PEACE-1 Investigators. Abiraterone plus prednisone added to androgen deprivation therapy and docetaxel in de novo metastatic castration-sensitive prostate cancer (PEACE-1): a multicentre, open-label, randomised, phase 3 study with a 2 × 2 factorial design. Lancet. 2022;399(10336):1695-1707. 10.1016/S0140-6736(22)00367-1 35405085

[39] Smith MR , Hussain M, Saad F, et al; ARASENS Trial Investigators. Darolutamide and survival in metastatic, hormone-sensitive prostate cancer. N Engl J Med. 2022;386(12):1132-1142. 10.1056/NEJMOA2119115 35179323 PMC9844551

[40] Seifert R , Emmett L, Rowe SP, et al Second version of the prostate cancer molecular imaging standardized evaluation framework including response evaluation for clinical trials (PROMISE V2). Eur Urol. 2023;83(5):405-412. 10.1016/j.eururo.2023.02.002 36935345

[41] Abdel-Aty H , O'Shea L, Amos C, et al The STAMPEDE2 trial: a site survey of current patterns of care, access to imaging and treatment of metastatic prostate cancer. Clin Oncol (R Coll Radiol). 2023;35(10):e628–35-e635. 10.1016/J.CLON.2023.07.009 37507278 PMC7616818

[42] Young S , Metser U, Sistani G, Langer DL, Bauman G. Establishing a provincial registry for recurrent prostate cancer: providing access to PSMA PET/CT in Ontario, Canada. Front Oncol. 2021;11:722430. 10.3389/FONC.2021.722430/BIBTEX 34408985 PMC8366560

[43] Hope TA , Truillet C, Ehman EC, et al 68Ga-PSMA-11 PET imaging of response to androgen receptor inhibition: first human experience. J Nucl Med. 2017;58(1):81-84. 10.2967/JNUMED.116.181800 27660139 PMC5209643

[44] Aggarwal R , Wei X, Kim W, et al Heterogeneous flare in prostate-specific membrane antigen positron emission tomography tracer uptake with initiation of androgen pathway blockade in metastatic prostate cancer. Eur Urol Oncol. 2018;1(1):78-82. 10.1016/J.EUO.2018.03.010 31100231

[45] Afshar-Oromieh A , Debus N, Uhrig M, et al Impact of long-term androgen deprivation therapy on PSMA ligand PET/CT in patients with castration-sensitive prostate cancer. Eur J Nucl Med Mol Imaging. 2018;45(12):2045-2054. 10.1007/s00259-018-4079-z 29980832 PMC6182397

[46] Marchetti L , Perrucci L, Pellegrino F, et al Diagnostic contribution of contrast-enhanced CT as compared with unenhanced low-dose CT in PET/CT staging and treatment response assessment of 18F-FDG–avid lymphomas: a prospective study. J Nucl Med. 2021;62(10):1372-1379. 10.2967/jnumed.120.259242 33712534

[47] Schöder H , Hope TA, Knopp M, et al; Members of the Working Group Considerations on integrating prostate-specific membrane antigen positron emission tomography imaging into clinical prostate cancer trials by National Clinical Trials Network Cooperative Groups. J Clin Oncol. 2022;40(13):1500-1505. 10.1200/JCO.21.02440 35015566 PMC9851697

[48] Feinstein AR , Sosin DM, Wells CK. The Will Rogers phenomenon. Stage migration and new diagnostic techniques as a source of misleading statistics for survival in cancer. N Engl J Med. 1985;312(25):1604-1608. 10.1056/NEJM198506203122504 4000199

